# Lung Cancer Therapy: The Role of Personalized Medicine

**DOI:** 10.3390/cancers17050725

**Published:** 2025-02-21

**Authors:** Raquel Ramos, Conceição Souto Moura, Mariana Costa, Nuno Jorge Lamas, Renato Correia, Diogo Garcez, José Miguel Pereira, Thomas Lindahl, Carlos Sousa, Nuno Vale

**Affiliations:** 1 PerMed Research Group, RISE-Health, Faculty of Medicine, University of Porto, Alameda Professor Hernâni Monteiro, 4200-319 Porto, Portugal; up202103841@up.pt (R.R.); carlos.sousa@unilabs.com (C.S.); 2 RISE-Health, Department of Pathology, Faculty of Medicine, University of Porto, Alameda Professor Hernâni Monteiro, 4200-319 Porto, Portugal; 3 Molecular Diagnostics Laboratory, Unilabs Portugal, Centro Empresarial Lionesa Porto, Rua Lionesa, 4465-671 Leça do Balio, Portugal; mariana.costa@unilabs.com (M.C.); nuno.lamas@external.unilabs.com (N.J.L.); 4 Pathology Laboratory, Unilabs Portugal, Rua Manuel Pinto de Azevedo 173, 4100-321 Porto, Portugal; conceicao.souto.moura@external.unilabs.com; 5 Anatomic Pathology Service, Pathology Department, Centro Hospitalar Universitário de Santo António (CHUdSA), Largo Professor Abel Salazar, 4099-001 Porto, Portugal; 6 Life and Health Sciences Research Institute (ICVS), School of Medicine, Campus de Gualtar, University of Minho, Rua da Universidade, 4710-057 Braga, Portugal; 7 Technology & Innovation Department, Unilabs Portugal, Rua Manuel Pinto de Azevedo 173, 4100-321 Porto, Portugal; renato.correia@unilabs.com (R.C.); diogo.garcez@unilabs.com (D.G.); 8 Radiology Department, Unilabs Portugal, Rua de Diogo Botelho 485, 4150-255 Porto, Portugal; jose.miguel.pereira@external.unilabs.com; 9 Unilabs Group Services, Succursale d’Unilabs, Laboratoire d’Analyses Médicales SA, Rue de Lausanne 15, 1201 Geneva, Switzerland; thomas.lindahl@unilabs.com; 10 Laboratory of Personalized Medicine, Department of Community Medicine, Health Information and Decision (MEDCIDS), Faculty of Medicine, University of Porto, Rua Doutor Plácido da Costa, 4200-450 Porto, Portugal

**Keywords:** lung cancer, genetic alterations, targeted therapy, personalized medicine, actionable mutations, survival

## Abstract

Lung cancer is the deadliest cancer worldwide, exhibiting the highest incidence rate among all cancer types. Poor outcomes often characterize this cancer as it is commonly diagnosed in advanced stages due to its unspecific symptoms. After diagnosis, the therapeutic choice is a crucial stage that profoundly affects patients’ survival. Treatment choices for lung cancer must be made carefully, acknowledging the histological type and genetic characteristics of the tumor. Non-small cell lung cancer, the most common and complex type, has a high mutational burden, making next-generation sequencing (NGS) essential for identifying specific mutations and guiding treatment. With several approved targeted therapies already available, this approach highlights the critical role of personalized medicine in lung cancer care. Despite the current therapeutic pipeline, research trying to develop new tailored drugs considering individual patient characteristics has evolved over the years. This article aims to outline the current therapeutic approach for each type of lung cancer and present the latest insights into emerging therapies, highlighting the role of personalized medicine in enhancing treatment outcomes and improving patients’ quality of life.

## Simple Summary

Lung cancer is the deadliest cancer globally, often diagnosed late due to nonspecific symptoms, leading to poor outcomes. Treatment choices, especially for non-small cell lung cancer (NSCLC), the most common type, require careful consideration of the tumor’s histology and genetic profile. NSCLC’s high mutational load makes next-generation sequencing (NGS) essential for identifying mutations and selecting targeted therapies. Personalized medicine, which tailors’ treatments based on individual genetic factors, is crucial in improving survival and quality of life. This article reviews the current therapeutic approaches for various lung cancer types and highlights advancements in emerging therapies that enhance treatment precision through personalization.

## 1. Lung Cancer: A Disease Introduction

Lung cancer (LC) ranks as the most prevalent and deadliest cancer in the world, with nearly 2.5 million new cases and 1.8 million deaths reported in 2022 [1].

Several risk factors increase the likelihood of developing this disease, including tobacco smoking, environmental and occupational exposure, family history of LC, and a history of other lung diseases. Among these, tobacco is the most significant risk factor, with approximately 80% of LC cases diagnosed in smokers [2,3]. Despite extensive knowledge of the risk factors related to this disease, LC is commonly diagnosed in advanced stages due to the non-specific symptoms—persistent cough, chest pain, dyspnoea, fatigue, and weight loss [4]. Consequently, LC is characterized by poor outcomes. The limited number of lung cancer screening (LCS) programs worldwide, with only three established in Europe, and the low adherence to them, contributes to delayed diagnoses and worse survival outcomes [5]. Currently, according to the most recent guidelines from the American Cancer Society, only patients considered to be at risk—between the ages of 50 and 80, and with at least a 20-pack-year history of smoking—are recommended to perform low-dose computer tomography (LDCT) for LCS [6]. However, broadening the selection criteria for more patients to be screened with LDCT is essential for early diagnosis and outcome improvement [6,7]. After a positive result in LDCT, an adequate tumor characterization for prognostication and treatment choice is essential due to the high heterogeneity typically associated with LC [8]. In fact, this tumor heterogeneity makes the therapy choice challenging, with the diversity of molecular profiles having the greatest impact on treatment options, as different molecular patterns result in different treatment responses even in patients with the same histological subtype. Despite the difficulties, the molecular diversity of LC presents a promising opportunity for personalized medicine, allowing more targeted therapies for each patient and improving their outcomes [8,9].

Therefore, considering the challenges associated with LC therapy and the continuous development in the field, this article will review the current therapeutic pipeline and the difficulties associated with drug resistance. The new insights and studies in this area will also be discussed, highlighting potential new drugs and reinforcing the importance of personalized medicine in LC treatment.

## 2. Current Therapies for LC

**Figure 1:**
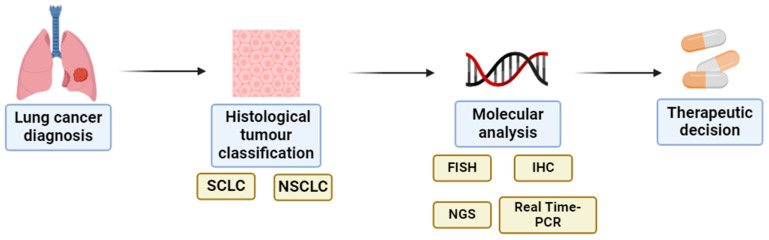
Steps from LC diagnosis to histological and molecular tumor classification for an adequate therapeutic decision. Laboratory techniques for molecular assessment of the tumor are also mentioned. Available online: http://biorender.com/ (accessed on 4 September 2024). SCLC—small-cell lung cancer; NSCLC—non-small-cell lung cancer; FISH—fluorescence in situ hybridization; IHC—immunohistochemistry; NGS—next-generation sequencing; Real-time PCR—real-time polymerase chain reaction.

As stated before, deciding which type of therapy will be applied to each lung tumor depends on numerous factors, such as histological subtype and molecular characteristics [9]. Specifically, after histological classification, non-small cell lung cancers (NSCLC) must undergo molecular analysis using several molecular techniques to identify potential genetic alterations that already have defined targeted therapies (Figure 1). Currently, all the recommended therapies for small cell lung cancer (SCLC) and NSCLC are listed in the National Comprehensive Cancer Network (NCCN), American Society of Clinical Oncology (ASCO), and European Society for Medical Oncology (ESMO) guidelines.

### 2.1. Small Cell Lung Cancer (SCLC)

SCLC represents around 10–15% of all primary lung tumors that are directly related to tobacco smoking. This type of LC rises from neuroendocrine cells of the basal bronchial epithelium and is characterized by poor prognosis, rapid growth, easy recurrence, and early widespread metastasis [10,11]. Considering the tumor’s fast growth, approximately 70% of patients with SCLC are diagnosed with extensive-stage SCLC (ES-SCLC), while just 30% present with limited-stage disease (LS-SCLC) [12]. SCLC is associated with a high mutation rate, namely the inactivation of two tumor suppressor genes—*TP53* and *RB1*. Nonetheless, despite these two genetic alterations being the most common, other genes may also be mutated in SCLC [13,14]. Table 1 shows the most commonly mutated genes in SCLC.

Despite all these known genetic alterations, how these tumors grow, metastasize, and respond to therapy remains poorly understood. Moreover, the most prevalent mutations are not actionable, and consequently, no targeted therapies are defined for SCLC, and the first treatment approach is decided based on tumor stage [14,15].

However, beyond the gene mutations presented in Table 1, SCLC classification remains a challenge, and the classification based on gene expression profiles is increasing. Recent studies have suggested a classification of SCLC into four subtypes considering the differential gene expression of ASCL1, NEUROD1, POU2F3, and YAP1 [16]. Specifically, the firsts to be identified were Achaete-scute homolog 1 (ASCL1) and neurogenic differentiation 1 (NEUROD1), which are neuronal transcription factors, and while ASCL1-high tumors were suggested to be associated with a high expression of neuroendocrine (NE) markers, NEUROD1-high tumors were associated with a lower expression of NE markers [17]. On the other hand, in ASCL1/NEUROD1 double-negative tumors—non-NE SCLC—an expression of POU2F3 was found—a marker of chemosensory tuft cells (brush cells in lung tumors) [18]. Finally, YAP1, a transcriptional regulator in the HIPPO growth signaling pathway, was found to be preferentially expressed in a subset of non-NE SCLC [13,19]. This classification holds significant promise for targeted therapy, as multiple molecules involved in the selection of emerging therapeutic agents demonstrate distinct distribution patterns across SCLC subtypes. For instance, Delta-like ligand 3 (DLL3), an inhibitor of the NOTCH signaling pathway, is regulated by ASCL1 and linked to the NE phenotype. Additionally, this classification advances immunotherapy, marking a major step forward in the personalized treatment of SCLC [17,20].

Apart from the molecular characteristics described, the immunogenic characteristics of SCLC arise as an interesting approach for therapy. Due to the high mutation rate associated with this type of LC, it is suggested that SCLC may be an immunogenic tumor, indicating a potential responsiveness to immunotherapy [21]. In fact, protein expression of the programmed death-ligand 1 (PD-L1)—a ligand of the immune checkpoint receptor programmed cell death protein 1 (PD-1)—has been found in multiple SCLC samples [22]. However, despite this finding, the clinical applicability of biomarkers of immunotherapy is still unknown because of the complexity of both the tumor and the microenvironment [23]. Nonetheless, several clinical trials have tested the efficacy of the blockade of some immune checkpoints, such as PD-1 and PD-L1, demonstrating that blocking their interaction increases the anticancer immunity in tumors, leading to a potential improvement of progression-free survival (PFS) and overall survival (OS) [24,25,26]. Due to the promising usefulness of these immunostimulatory targets, researchers are trying to understand the utility of other biomarkers. For example, a study investigated the expression level of B7-family ligands, such as B7-H3 and B7-H4, in SCLC samples and their impact on the anti-tumor immune response. The results showed a predominant expression of B7-H3 and a limited cytotoxic immune response, demonstrating the importance of evaluating B7-H3 blockers and pro-inflammatory therapies [12,27]. Other immune checkpoints, such as cytotoxic T-lymphocyte-associated antigen 4 (CTLA-4), are under study for SCLC treatment. Therefore, for CTLA-4, the immune checkpoint inhibitors (ICIs) ipilimumab and tremelimumab are under investigation [28]. Among the PD-1 inhibitors, nivolumab and pembrolizumab [29,30,31,32,33,34], and the PD-L1 inhibitors atezolizumab and durvalumab [21,31], are already approved by the U.S. Food and Drug Administration (FDA) for immunotherapy in SCLC, being currently recommended for disease therapy, according to NCCN guidelines. The first antibody approved was nivolumab, in 2018, as a third-line treatment for recurrent SCLC. Later, in 2019, atezolizumab was approved for extensive staged SCLC as a first-line treatment in combination with chemotherapy. Recently, durvalumab was also approved in combination with chemotherapy as a first-line treatment for extensive staged SCLC [12].

Moreover, the efficacy of some of these ICIs was also tested in combined therapy, but nonetheless, the results were not encouraging. Specifically, a phase 1/2 trial—CheckMate 032 trial (NCT01928394)—was performed to evaluate the efficacy of nivolumab alone or in combination with ipilimumab in recurrent SCLC. However, OS was similar between groups, and the toxicities were higher with combination therapy [35]. Concomitantly, a double-blind phase III trial—CheckMate 451 (NCT02538666)—concluded that maintenance therapy with nivolumab plus ipilimumab did not prolong OS for patients with extensive SCLC [36].

Currently, according to the most recent update of NCCN guidelines [37], all patients with SCLC must receive systemic therapy [38]; however, the first treatment approach is decided based on tumor stage. In non-metastatic patients, the intent is to achieve control of tumor growth and reduce the risk of metastatic dissemination with local treatment. For this, in very early limited-stage SCLC (LS-SCLC), surgery is the first approach to control thoracic disease. Additionally, patients not eligible for surgery may be candidates for stereotactic ablative radiotherapy (SABR) [39,40,41]. Nonetheless, postoperative systematic treatment with chemotherapy (cisplatin–etoposide/carboplatin–etoposide) and radiotherapy (RT) is needed [14,42].

However, in patients with non-metastatic locally advanced SCLC, which involves the mediastinal and hilar nodes, radiotherapy with concomitant chemotherapy (cisplatin–etoposide) is the most well-established treatment approach. Concomitantly, prophylactic cranial irradiation (PCI) is also part of the standard management in most patients with non-metastatic SCLC who respond to initial treatment to reduce the risk of brain metastasis [43]. In metastatic SCLC, chemotherapy combined with immunotherapy is the best treatment approach. Complementary, subsequent consolidative thoracic radiotherapy can be beneficial with a complete or good response to systemic therapy before immunotherapy, especially with residual thoracic disease and low-bulk extrathoracic metastatic disease. Nonetheless, when brain metastasis exists, brain RT can be administrated either before or after systemic therapy [21,31,44,45].

Beyond the therapeutic recommendations previously described, the investigation of new therapeutic options for SCLC continues. As stated before, due to the relation between DLL3 and ASCL1, and since it is frequently upregulated in neuroendocrine subtypes in SCLC, DLL3 has emerged as a promising therapeutic target in this tumor subtype [46]. Recently, Tarlatamab, a bispecific T-cell engager targeting both delta-like ligand 3 (DLL3) of cancer cells and CD3 on T-cells, was approved by FDA for ES-SCLC with disease progression on or after platinum-based chemotherapy [47,48]. This approval was based on a phase 2 open-label, multicenter, multi-cohort study (DeLLphi-301), which showed durable objective responses and promising survival outcomes on patients with previously treated SCLC [49]. Earlier, a phase 1 trial showed encouraging antitumor activity and controllable safety in the same type of patients [50].

In addition, a recent phase 3 study (ADRIATIC) demonstrated that adjuvant therapy using durvalumab increased patients’ survival and progression-free survival among patients with LC-SCLC [51]. This therapy was already approved by the FDA [52].

**Figure 2:**
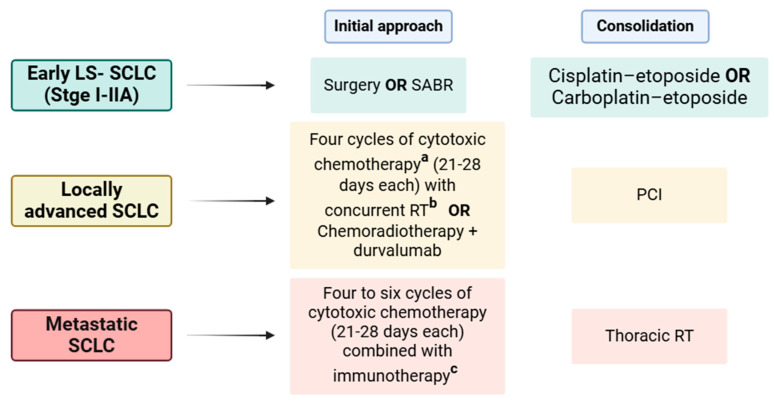
Therapeutic scheme for different stages of SCLC. The initial recommended approach and the consolidation treatment are indicated. Specification of the preferred therapeutic regimen is also described [37,52]. Available online: http://biorender.com/ (accessed on 18 February 2025). SABR—stereotactic ablative radiotherapy; PCI—prophylactic cranial irradiation; RT—radiotherapy; ^a^ cisplatin 75 mg/m^2^ day 1 and etoposide 100 mg/m^2^ days 1, 2, 3; cisplatin 60 mg/m^2^ day 1 and etoposide 120 mg/m^2^ days 1, 2, 3; ^b^ 45 Gy in 3 weeks; ^c^ carboplatin day 1 and etoposide 100 mg/m^2^ days 1, 2, 3 and atezolizumab 1200 mg day 1 every 21 days × 4 cycles followed by maintenance atezolizumab 1200 mg day 1, every 21 days; carboplatin day 1 and etoposide 100 mg/m^2^ days 1, 2, 3 and atezolizumab 1200 mg day 1 every 21 days × 4 cycles followed by maintenance atezolizumab 1680 mg day 1, every 28 days; carboplatin day 1 and etoposide 80–100 mg/m^2^ days 1, 2, 3 and durvalumab 1500 mg day 1 every 21 days × 4 cycles followed by maintenance durvalumab 1500 mg day 1 every 28 days; cisplatin 75–80 mg/m^2^ day 1 and etoposide 80–100 mg/m^2^ days 1, 2, 3 and durvalumab 1500 mg day 1 every 21 days × 4 cycles followed by maintenance durvalumab 1500 mg day 1 every 28 days.

Figure 2 explains in detail the therapeutic approaches already approved in each stage of SCLC.

### 2.2. Non-Small Cell Lung Cancer (NSCLC)

NSCLC is the most common type of LC, comprising approximately 85% of all LC cases [11,53]. In addition, this LC type can be divided into three main subtypes—adenocarcinoma (LUAD), large cell carcinoma, and squamous cell carcinoma—where LUAD is the most frequent one (≈40% of all NCLC), being characterized by larger cells and a glandular pattern [7,54,55]. NSCLC is characterized by a wide range of genetic alterations, some of which are actionable mutations that serve as promising targets for therapies [56,57]. In fact, the identification of histological and/or molecular NSCLC subtypes is crucial for determining the therapy approach, defining a personalized treatment, and improving prognosis [58]. Therefore, the current knowledge about the NSCLC landscape and its genetic modifications contributes to personalized and precision medicine applications [59,60,61].

As previously mentioned, the molecular analysis is conducted using various molecular techniques (Figure 1), and the NCCN for NSCLC recommends routine genomic biomarker testing in all patients with advanced NSCLC. The genetic changes stated as the most important ones to be tested are mutations and/or amplifications in *EGFR*, *KRAS*, *BRAF*, *ERBB2*, and *MET*, as well as *ALK*, *ROS1*, *NTRK1,2,3*, and *RET* rearrangements [62]. In addition, as in SCLC, the immunostimulatory targets are also important for NSCLC therapeutic decisions. Specifically, the current guidelines recommend PD-L1 expression evaluation by immunochemistry for all patients with NSCLC for immunotherapy consideration. Nonetheless, in the case of a PD-L1-positive result (PD-L1 ≥ 50%), this immune approach is only advisable for patients without *EGFR* exon 19 deletion, L858R mutation, or *ALK* rearrangements, as these are less responsive to immune checkpoint inhibitors. Despite the efficacy of immunotherapy being generally lower in the range 1% ≤ PD-L1 ≥ 49%, these patients still benefit from immune checkpoint inhibitors like pembrolizumab, nivolumab, and ipilimumab, either alone or in combination with chemotherapy. All these biomarker tests are important, as a known alteration in one of them implies a specific treatment for patients using targeted therapies [62,63,64]. Table 2 shows the biomarkers tested in NSCLC diagnosis and the approved targeted therapies for each.

Contrary to SCLC, the therapeutic approach of NSCLC is more complex and requires more tests to understand the most adequate therapy. For stage I NSCLC, surgery is the first therapeutic choice with post-operative radiation or chemotherapy to reduce the chances of disease recurrence. In stages II and III of the disease, surgery may be supported by chemotherapy, radiotherapy, or immunotherapy. In more advanced stages (stage IV), where tumors have disseminated throughout the body and surgery cannot achieve complete resection, searching for specific mutations is necessary to apply targeted therapies. In case of the absence of actionable oncogenic drivers, patients should be treated with chemotherapy with platinum doublets [64,67].

Despite the recommendation of chemoradiotherapy used in stages II and III, there are already clinical trials proving the efficacy of immunochemotherapy as a perioperative treatment for NSCLC. Specifically, phase 2 studies showed that neoadjuvant nivolumab alone or with chemotherapy have promising results in survival and safety outcomes in resectable NSCLC [68,69,70]. Complementarily, a phase 3 study proved that patients with stages IB to IIIA resectable NSCLC treated with neoadjuvant nivolumab plus chemotherapy have higher event-free survival. Another phase 3 trial confirmed the previous study, demonstrating that patients with resectable stage IIA to IIIB NSCLC receiving neoadjuvant nivolumab plus chemotherapy have a longer event-free survival than those receiving chemotherapy alone. Therefore, these results show that neoadjuvant immunochemotherapy is a valuable option for NSCLC treatment, even in more early stages (IB), being already approved in the United States and the European Union [71,72]. In addition, using adjuvant immunotherapy after surgery followed by adjuvant chemotherapy has also been demonstrated to be a potentially new treatment approach for resected NSCLC [73,74].

**Figure 3:**
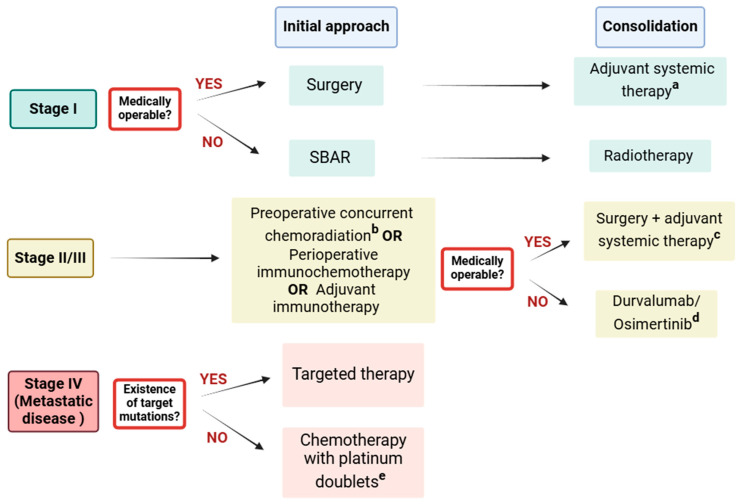
Therapeutic scheme for different stages of NSCLC. The initial recommended approach and the consolidation treatment are indicated. Specification of the preferred therapeutic regimen is also described [71,72,73,74,75]. Available online: http://biorender.com/ (accessed on 18 February 2025). ^a^ Osimertinib 80 mg daily for 3 years; ^b^ preferred therapy (nonsquamous): carboplatin on day 1, pemetrexed 500 mg/m^2^ on day 1 every 21 days for 4 cycles; concurrent thoracic RT OR cisplatin 75 mg/m^2^ on day 1, pemetrexed 500 mg/m^2^ on day 1 every 21 days for 3 cycles; concurrent thoracic RT OR paclitaxel 45–50 mg/m^2^ weekly; carboplatin, concurrent thoracic RT OR cisplatin 50 mg/m^2^ on days 1, 8, 29, and 36; etoposide 50 mg/m^2^ days 1–5 and 29–33; concurrent thoracic RT; preferred therapy (squamous): paclitaxel 45–50 mg/m^2^ weekly; carboplatin, concurrent thoracic RT OR cisplatin 50 mg/m^2^ on days 1, 8, 29, and 36; etoposide 50 mg/m^2^ days 1–5 and 29–33; concurrent thoracic RT; ^c^ preferred therapy (nonsquamous): cisplatin 75 mg/m^2^ day 1, pemetrexed 500 mg/m^2^ day 1 every 21 days for 4 cycles; preferred therapy (squamous): cisplatin 75 mg/m^2^ day 1, gemcitabine 1250 mg/m^2^ days 1 and 8, every 21 days for 4 cycles OR cisplatin 75 mg/m^2^ day 1, docetaxel 75 mg/m^2^ day 1 every 21 days for 4 cycles; ^d^ durvalumab 10 mg/kg IV every 2 weeks or 1500 mg every 4 weeks for up to 12 months; osimertinib 80 mg once daily until disease progression if EGFR exon 19 deletion or L858R; ^e^ preferred therapy (nonsquamous): (carboplatin or cisplatin) + pemetrexed + pembrolizumab OR cemiplimab + pemetrexed + (carboplatin or cisplatin); preferred therapy (squamous): carboplatin + (paclitaxel or albumin-bound paclitaxel) + pembrolizumab OR cemiplimab + paclitaxel + (carboplatin or cisplatin).

Figure 3 explains the recommended therapeutic strategy for each NSCLC stage.

**Figure 4:**
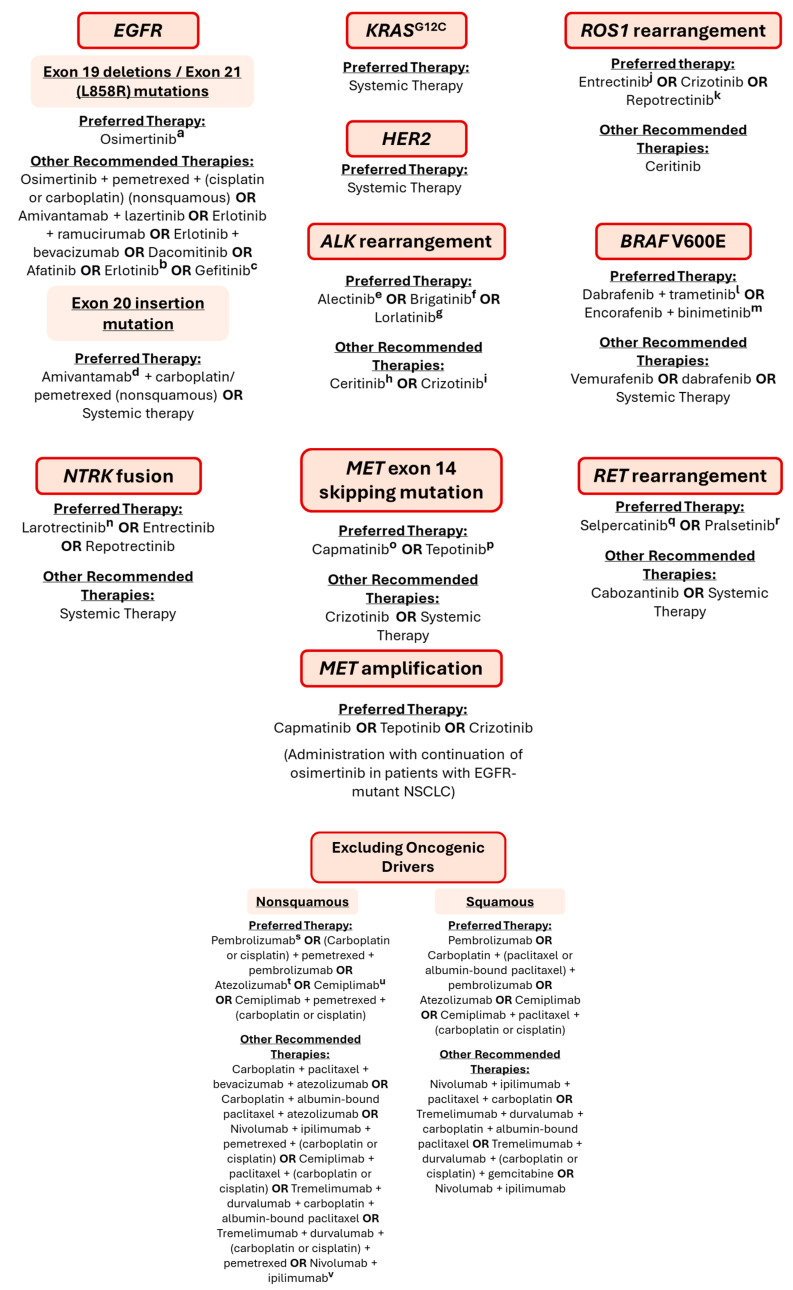
Preferred and alternative targeted therapies recommended for each biomarker and for no oncogenic driver mutations scenarios [75]. The recommended dosages according to the FDA for the main drugs are listed. ^a^ 80 or160 mg/day for a median of 260 and 171 days, respectively *; ^b^ 150 mg/daily *; ^c^ 250 mg/daily *; ^d^ 1050 mg (body weight < 80 kg) or 1400 mg (body weight ≥ 80 kg) once weekly for 4 weeks *; ^e^ 600 mg orally twice daily with food for 2 years or until disease recurrence or unacceptable toxicity *; ^f^ 90 mg orally once daily for the first 7 days, then increase to 180 mg orally once daily *; ^g^ 100 mg once daily *; ^h^ 750 mg orally once daily *; ^i^ 250 mg orally twice daily until disease progression or unacceptable toxicity *; ^j^ 600 mg orally once daily *; ^k^ 160 mg orally once daily with or without food for 14 days, then increased to 160 mg twice daily, until disease progression or unacceptable toxicity *; ^l^ dabrafenib 150 mg (two 75 mg capsules) orally twice daily in combination with trametinib 2 mg orally once daily *; ^m^ encorafenib 450 mg orally once daily and binimetinib 45 mg orally twice daily *; ^n^ 100 mg orally twice daily *; ^o^ 400 mg orally twice daily *; ^p^ 450 mg orally once daily *; ^q^ 120 mg orally twice daily (<50 kg) or 160 mg orally twice daily (≥50 kg) *; ^r^ 400 mg orally once daily *; ^s^ 200 mg every 3 weeks or 400 mg every 6 weeks, administered by intravenous infusion *; ^t^ 840 mg every 2 weeks, 1200 mg every 3 weeks, or 1680 mg every 4 weeks, administered intravenously *; ^u^ 350 mg every 3 weeks, intravenously *; ^v^ 360 mg nivolumab every 3 weeks with ipilimumab 1 mg/kg every 6 weeks *; * According to FDA recommended dosage (https://www.fda.gov/, accessed on 1 October 2024).

Considering the most advanced stage of NSCLC and the targeted therapies available, Figure 4 describes in detail the therapeutic pipeline recommended for each genetic alteration and according to the level of PD-L1 expression.

#### Drug Resistance—The Second-Line Therapy

Despite the extensive range of therapeutic options already developed for NSCLC treatment, which improved the PFS and the OS of patients, most of them develop resistance to the first-line therapy after nearly one year [76]. Consequently, patients should be closely monitored to track disease progression and adjust the therapy approach in case of loss of therapeutic response and resistance development due to molecular alterations. The detection of genetic alterations involves laboratory techniques, namely NGS. In addition, other techniques can be used to detect some alterations: qPCR for detecting *EGFR*, *BRAF*, and *KRAS* mutations, and IHC or FISH for *ALK* and *ROS* rearrangements and *MET* gene amplification [77,78]. This molecular analysis usually requires a new biopsy (rebiopsy), but a liquid biopsy offers an excellent alternative when this procedure is not possible. In some cases, a positive result in liquid biopsy for some mutations, T790M for example, avoids the need for a rebiopsy. Nonetheless, in specific instances like histological type transformation, liquid biopsy may not be the best approach, and a fresh tissue biopsy becomes essential [7].

According to the most recent NCCN guidelines, in cases of tumor progression, systemic therapy is recommended as an alternative to targeted drugs. This alternative includes the following preferred regimens: pembrolizumab/(carboplatin or cisplatin)/pemetrexed, cemiplimab/pemetrexed/(carboplatin or cisplatin), or carboplatin/pemetrexed for patients with no contraindications to PD-1/PD-L1 inhibitors. Local treatment using SABR or surgery is also an option given by the international guidelines [75]. Nonetheless, some mutations already have an alternative target therapy. In the *EGFR* gene, T790M is a common mutation that is responsible for resistance to erlotinib and gefitinib. Consequently, in patients first treated with these drugs and who develop this specific mutation, osimertinib should be administered as an alternative therapy. Patients with an *EGFR* exon 20 insertion mutation who had progression on platinum chemotherapy receive a subsequent therapy with amivantamab [75,76,79]. For *HER2* and *KRAS* genetic alterations, when the first therapeutic approach fails (systemic therapy), trastuzumab deruxtecan and sotorasib or adagrasib, respectively, are recommended as subsequent therapy. However, *KRAS* second-line therapy is recommended if no previous *KRAS* G12C-targeted therapy exists. Regarding *ALK* rearrangements, lorlatinib is an option for resistant mutations, such as *ALK* G1202R and L1196M. In the case of crizotinib resistance, a subsequent therapy with alectinib, brigatinib, ceritinib, or lorlatinib is suggested [75,80].

## 3. Emerging Treatment Strategies for LC

Despite the current treatment approaches for LC, research about new possible drugs continues to enhance therapeutic options focused on personalized medicine to improve patient outcomes. Johnson & Johnson Innovative Medicine is one of the pharmaceutical companies that promotes the creation and study of new therapeutic approaches, including treatments for LC. As previously mentioned, amivantamab-vmjw is a drug developed by this company that was already approved by the FDA in combination with chemotherapy for the first-line treatment of patients with locally advanced or metastatic NSCLC with *EGFR* exon 20 insertion mutations based on positive results from the randomized, open-label phase 3 PAPILLON study (NCT04538664) [75,81]. Nonetheless, other ongoing clinical trials are studying the therapeutic efficacy of amivantamab-vmjw with chemotherapy and/or other drugs for LC treatment. Along with testing of new combined therapy, clinical trials investigating the most advantageous route of drug administration are also being carried out [82]. Table 3 summarizes these clinical trials.

Beyond these several clinical trials performed by Johnson & Johnson Innovative Medicine, other research groups aim to improve the therapeutic options of LC patients. Saracatinib (AZD0530) is a dual Src/Abl inhibitor initially developed by AstraZeneca that, despite the toxicity issues seen in several cancers, demonstrated encouraging results in LC treatment in a phase II clinical trial. The results of this trial showed minimal side effects, partial therapeutic response, and tumor stabilization in NSCLC patients, also raising the hypothesis that there probably exists a subset of saracatinib-responsive NSCLC [90,91]. In accordance with these findings, another research group performed pre-clinical trials using NSCLC cell lines carrying the *EGFR* T790M mutations and demonstrated that these cells have a better therapeutic response to the combined treatment with saracatinib and cetuximab [92].

Immunotherapy is another area that is highly studied, as a small proportion of patients can benefit from it, and therapeutic resistance occurs due to the complexity and diversity of the tumor microenvironment (TME). Consequently, new approaches to improve this therapy are being studied. One example is tumor-derived exosomes (TEXs) [93]. These exomes carry a variety of cargoes and have both immunosuppressive and immunostimulatory action by delivering molecular signals to immune cells in several tumours, including LC [94,95]. In addition, TEXs can efficiently deliver tumor antigens to dendritic cells (DCs), making them excellent candidates to be applied in LC immunotherapy, being used as self-antigens carriers and to stimulate the immune response and enhance the anti-tumor effects [93]. The use of TEXs for DC vaccine development is also a promising approach under study [96].

Despite these efforts to improve LC treatment and the availability of drugs already approved by international guidelines, access to many of these drugs varies across countries. In Portugal, drugs must receive approval from INFARMED (National Authority for Medicines and Health Products) to be used in clinical routine and, sometimes, this bureaucratic process takes time. This issue is not exclusive to Portugal, as many other countries experience slow and lengthy drug approval processes. This delay affects patient care by limiting the availability of certain critical medications, leading to treatment delays or the use of suboptimal and consequently less effective therapies.

Moreover, another aspect that must be taken into consideration is drug toxicities, not only at the time of therapy [97] but also during the decision-making process prior to treatment, and the evaluation of each patient’s metabolic characteristics and prediction of some potential toxicities that a specific drug may cause. This process is also an important aspect of personalized medicine in LC treatment that must be carefully considered and integrated into clinical practice to ensure the best therapeutic choices and proper drug dosage adjustments for each patient.

## 4. Conclusions

Several characteristics contribute to the heterogeneity of LC, with the molecular signature being one of the most significant. Along with histological classification, the molecular characterization of NSCLC is crucial for therapeutic decisions. In fact, NSCLC demonstrates more complexity in therapeutic decisions compared to SCLC, as NSCLC is defined by several genetic alterations that are eligible for approved targeted therapies. This possibility of administrating targeted therapies to each patient according to the tumor’s molecular characteristics makes LC an ideal disease for personalized medicine, improving patients’ outcomes. Consequently, despite the current therapeutic pipeline recommended by NCCN international guidelines, ongoing research to improve the therapeutic options for LC patients remains indispensable. Pharmaceutical companies and research groups are committed to discovering new drugs, always focused on personalized medicine to provide an adapted treatment for each patient and improve their survival. Nonetheless, despite these efforts, it is still crucial to accelerate the drug approval process by the authorities in each country, as well as to take into account the toxicities and tolerances of each patient to various treatments. Addressing all these challenges while recognizing the importance of continuing to incorporate personalized medicine into clinical practice is essential to achieving meaningful progress and improved outcomes for lung cancer patients.

## Figures and Tables

**Table 1:** Most frequently altered genes in SCLC with the respective frequency and the specific genetic alteration [14].

Gene	Frequency (%)	Genetic Alteration
*TP53*	89	Inactivating mutation/Deletion
*RB1*	64	Inactivating mutation/Deletion
*KMT2D*	13	Inactivating mutation/Deletion
*PIK3A*	7	Activating mutation
*PTEN*	7	Inactivating mutation/Deletion
*NOTCH1*	6	Inactivating mutation
*CREBBP*	5	Inactivating mutation/Deletion
*FAT1*	4	Inactivating mutation/Deletion
*NF1*	4	Inactivating mutation/Deletion
*APC*	4	Inactivating mutation/Deletion
*EGFR*	4	Activating mutation
*KRAS*	3	Activating mutation

**Table 2:** Tested biomarkers in NSCLC for therapeutic decision with the respective frequency and the most common genetic alterations [63,65,66].

Biomarker	Frequency (%)	Typical Genetic Alterations
*EGFR*	10–15 (59–50 Asian)	Exon 19 deletions/Exon 21 (L858R) substitution mutations
		T790M mutation
		Exon 20 insertion mutations
*ALK*	5	Fusion
*ROS1*	1–2	Fusion
*NTRK 1/2/3*	0.23–32	Fusion
*BRAF*	2	V600E mutation
*RET*	1–2	Fusion
*MET*	3	Exon 14 skipping alterations
*KRAS*	12	G12C mutation
*HER2*	2–5	Exon 20 insertions
PD-L1	23–28	High expression

**Table 3:** Ongoing clinical trials testing new therapeutic combinations with amivantamab-vmjw and alternative routes of administration for LC treatment. Clinical trials are identified by the ClinicalTrials.gov Identifier (accessed on 18 September 2024).

Targeted Genetic Alterations	Route of Drug Administration Tested	Therapy Tested	Clinicaltrials.gov Identifier	Ref
*EGFR* ex19del or L858R who had disease progression on or after treatment with osimertinib	-	amivantamab-vmjw + lazertinib + chemotherapy	Phase 3 MARIPOSA-2 (NCT04988295)	[83]
Advanced NSCLC with *EGFR* mutations	-	amivantamab-vmjw + lazertinib AND lazertinib as a monotherapy	Phase 1/1b CHRYSALIS-2 (NCT04077463)	[84]
Locally advanced or metastatic NSCLC	-	amivantamab-vmjw + capmatinib	Phase 1/2 METalmark (NCT05488314)	[85]
Locally advanced or metastatic NSCLC	-	amivantamab-vmjw + cetrelimab	Phase 1/2 PolyDamas (NCT05908734)	[86]
Advanced solid malignancies	Subcutaneous administration	amivantamab-vmjw	Phase 1 PALOMA (NCT04606381)	[87]
*EGFR* Ex19del or L858R-mutated advanced NSCLC	Subcutaneous administration	amivantamab-vmjw + lazertinib	Phase 2 PALOMA-2 (NCT05498428)	[88]
*EGFR*-mutated advanced NSCLC who progressed after osimertinib and platinum-based chemotherapy	Subcutaneous Versus Intravenous administration	amivantamab-vmjw + lazertinib	Phase 3 PALOMA-3 (NCT05388669)	[89]

## Data Availability

Not applicable.
